# Compactly representing massive terrain models as TINs in CityGML

**DOI:** 10.1111/tgis.12456

**Published:** 2018-09-26

**Authors:** Kavisha Kumar, Hugo Ledoux, Jantien Stoter

**Affiliations:** ^1^ 3D Geoinformation Delft University of Technology Delft The Netherlands

## Abstract

Terrains form an important part of 3D city models. GIS practitioners often model terrains with 2D grids. However, TINs (Triangulated Irregular networks) are also increasingly used in practice. One such example is the 3D city model of the Netherlands (3DTOP10NL), which covers the whole country as one massive triangulation with more than one billion triangles. Due to the massive size of terrain datasets, the main issue is how to efficiently store and maintain them. The international 3D GIS standard CityGML allows us to store TINs using the Simple Feature representation. However, we argue that it is not appropriate for storing massive TINs and has limitations. We focus in this article on an improved storage representation for massive terrain models as TINs. We review different data structures for compactly representing TINs and explore how they can be implemented in CityGML as an ADE (Application Domain Extension) to efficiently store massive terrains. We model our extension using UML, and XML schemas for the extension are automatically derived from these UML models. Experiments with massive real‐world terrains show that, with this approach, we can compress CityGML files up to a factor of ~20 with one billion+ triangles, and our method has the added benefit of explicitly storing the topological relationships of a TIN model.

## 1 Introduction

The use of 3D city models for urban planning and management has increased in recent years. Several cities like Rotterdam, Brussels, and Berlin have already created 3D city models for use in different applications such as noise mapping, estimating the energy demand of buildings, and calculating building rooftop solar irradiation (Biljecki, Stoter, Ledoux, Zlatanova, & Çöltekin, 2015). However, in practice these applications are mostly centered around buildings; other terrain features like vegetation, roads, and water bodies are often ignored. Formal specifications for modeling buildings in 3D space are often more prominently defined than other urban features. For example, in the international 3D GIS standard CityGML, the concept of LODs (Levels Of Detail) is very well established for buildings and bridges, but is vague in case of terrains and land use (OGC, 2012).

Over the last few decades, grids and TINs (Triangulated Irregular Networks) have become the two most popular models for representing terrains. GIS practitioners often model terrains as grids. However, grids have several shortcomings. First, they cannot be used to represent terrains with vertical walls and overhangs, which are quite common in cities (we give precise definitions of these in Section 2). Second, grids, being restricted to 2.5D, do not conform well to the variability in terrain complexity. This might result in loss of sample points, which could be important for spatial analysis such as points representing balconies, dormers, chimneys, vertical walls, and banks of canals (Fisher, 1997). Another disadvantage is that grids can be very large for fine‐resolution terrains (Fisher, 1997). For instance, in case of 3D grids, the size of voxels (3D pixels) increases as the resolution of data increases, which requires more storage space (Stoter & Zlatanova, 2003). On the other hand, TINs have numerous benefits. In a TIN, the local density of points can be altered based on the variations in height of the original terrain (Kumler, 1994). For example, areas of detailed relief can be represented in a TIN with a denser triangulation than areas with a smooth relief (Kumler, 1994). Another advantage is that the points describing balconies, dormers, chimneys, and vertical walls can be well represented as constraints in a TIN (Kumler, 1994). However, storing TINs is more complicated than storing grids, as it requires not only storing the TIN geometry but also efficiently storing and ing the topological relationships between the triangles. A terrain can be stored either as one massive TIN with continuous elevation values or as a constrained TIN with 3D objects like buildings, roads, and vegetation as constraints in the triangulation.

**Figure 1:**
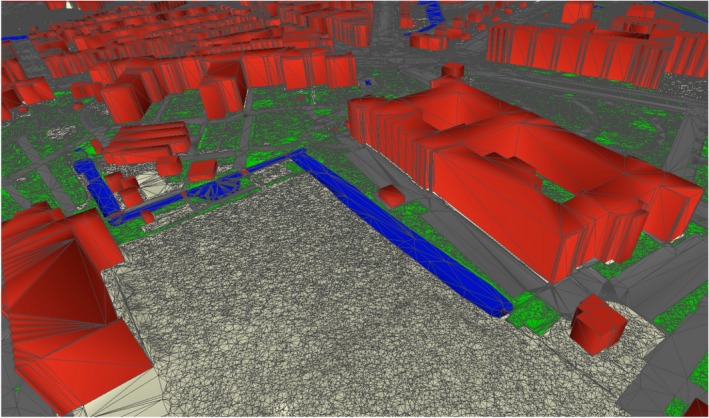
Snapshot of 3DTOP10NL dataset of a part of Delft, the Netherlands. Note that the terrain is one massive TIN with buildings, roads, water bodies, and other features. CityGML requires ~700 GB of storage space just for storing the 3DTOP10NL terrain geometry

CityGML supports the storage of DTMs (Digital Terrain Models) as TINs but it is not efficient for storing massive TINs. Generally, the number of triangles in a TIN is roughly twice the number of vertices used in triangulation (De Berg, Van Kreveld, Overmars, & Schwarzkopf, 2000). The CityGML datasets can become very large for massive TINs because of the redundancy in the underlying data structure, which greatly hinders web‐based rendering and exchange of data. Moreover, there is very little topological information stored, which prevents us from efficiently using the datasets for analysis. For instance, 3DTOP10NL (Kadaster, 2015), the 3D city model of the Netherlands, covers the whole country (including buildings, roads, water bodies, and bridges) as one massive triangulation with more than one billion triangles (Figure 1). CityGML requires a file size of ~700 GB just to store the geometry of the 3DTOP10NL terrain dataset (without any topological information).

Therefore, the main focus of this article is to develop an improved representation for storing massive terrains as TINs in the context of 3D city models. This article is an actual implementation of and extension to the ideas that we proposed in the initial phase of the research (Kumar, Ledoux, & Stoter, 2016a, 2016b). In this article, we review different data structures for compactly representing TINs, and explore how they can be implemented in GML/CityGML to efficiently store massive TINs. The research is not limited to model terrains as 2.5D TINs. It also includes vertical walls, overhangs, and constraints in the terrain model (see Section 2). Three existing compact TIN data structures, namely *Indexed triangles* (Ravada, Kazar, & Kothuri, 2009), *TriStrips* (Speckmann & Snoeyink, 2001), and *Stars* (Ledoux, 2015), are introduced as new geometry types in the GML geometry model for representing TINs. These new geometry types are extended to CityGML as an ADE (Application Domain Extension) for compactly representing massive TIN terrains (see Section 3). We model the extension using UML (Unified Modeling Language). XML schemas for the extension are automatically derived from these UML models. We made a prototype to implement these TIN data structures in CityGML datasets. We tested our proposed CityGML extension with several real‐world datasets and we report on the compression factors achieved in Section 4. Our approach allows us to compress up to a factor of ~20 with massive real‐world terrain datasets. For example, the storage space required for the 3DTOP10NL terrain in a CityGML file is reduced from ~700 GB to nearly ~40 GB. Moreover, our method has the added advantage of explicitly storing the topological relationships of a TIN model. We close the article with conclusions and future work in Section 5.

## 2 State‐of‐the‐art in modeling terrains with TINs

Terrain (Latin *Terra* meaning Earth) in simple terms refers to the lay of the land described in terms of elevation, slope, or other attributes of the landscape (Wikipedia, 2017). Modeling the terrain surface with precision has always been a challenge for geo‐researchers. The irregular nature of the surface makes it difficult to depict the true model of a terrain. In this section, we provide an overview of different TIN representations used for modeling terrains. Several data structures have been proposed in different domains to represent and store TINs; they exhibit data redundancy and also store information for maintaining the adjacency relationships. We review different TIN data structures that can be integrated efficiently in the GML3 geometry model and extended to CityGML for representing massive terrains.

### 2.1 Representation of terrains

**Figure 2:**
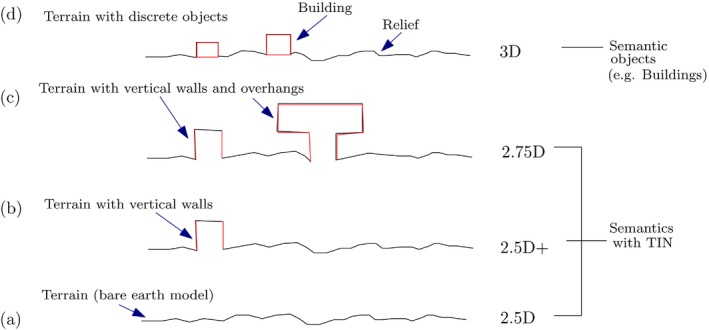
Different TIN representations for modeling terrains considered in this research. Semantics are attached to the entire TIN in 2.5D/2.5D+/2.75D and to the discrete objects (e.g. buildings) embedded in the TIN in 3D

A terrain is usually modeled as a grid of elevation values or as a TIN. These are also referred to as field representations in GIS (Kumler, 1994; Cova & Goodchild, 2002). A *field* is a model of spatial variation of an attribute over a spatial domain (Ledoux, 2017). Fields are generally used to represent continuous geographical phenomena such as the elevation of a terrain, surface temperature, and so on (Ledoux, 2017; Cova & Goodchild, 2002). A terrain can be modeled as a field, by a function *f*(*x*, *y*) mapping each (*x*, *y*) location in the spatial domain to an elevation value (*z*) [i.e. *z* = *f*(*x*, *y*)] (Figure 2a).

**Figure 3:**
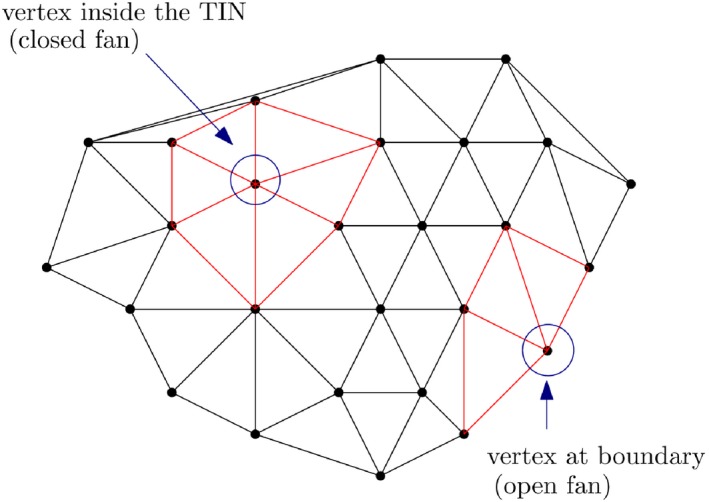
2‐Manifold TIN. Each edge of the TIN is incident only to one or two triangles of the TIN

Modeling terrains by storing only one elevation value (*z*) for any (*x*, *y*) location is referred to as “2.5D” (Figure 2a). Topologically, the surface depicted by a TIN is a *2‐manifold* (i.e., each edge of the TIN is incident to only one or two triangles) and the triangles incident to a vertex form either a closed or an open fan (Gotsman, Gumhold, & Kobbelt, 2002) (Figure 3).

However, it is not possible to represent features like vertical walls, roof overhangs, caves/tunnels, and overfolds like balconies and dormers with 2.5D field models. For instance, 3DTOP10NL terrain data has vertical walls. Modeling it in 2.5D will result in loss of information points representing the vertical walls. Therefore, we focus on geometrical representations which extend the field‐based 2.5D model to handle such features. In Figure 3b, an example is shown where a location (*x*, *y*) has more than one elevation value (*z*) to model the vertical walls of natural or man‐made objects like buildings. It is a so‐called “2.5D+” model, which is topologically equivalent to a 2.5D model as it is still a 2‐manifold (Penninga, 2008).

The ISO 19107:2003 Spatial Schema (ISO, 2003) standard defines GM_TIN geometry type for representing TIN models, which in theory should allow vertical triangles in a TIN and therefore can be referred to as a 2.5D+ data structure. Features like balconies, and overhangs of rocks and roof surfaces, are not covered by these models and are described using 2.75D models (Tse & Gold, 2004; Gröger & Plümer, 2005). A “2.75D” model is a 2.5D+ model extended to model any 2‐manifold surface with features like balconies and overhangs (Figure 2). These models are described in the context of TINs and not grids. They are sufficient for applications like visualization and watershed modeling (Lyon, 2003).

However, for some applications, even 2.5D+ and 2.75D models have limitations. For instance, applications estimating population and building energy demand using 3D city models require computing the volume of buildings (Biljecki et al., 2015), which is not possible to calculate using these terrain models. To compute the volume of a building, it should be closed at the base (i.e., modeled as a solid). Based on the above argument, we refer to the 3D model of a terrain as a 2.5D+/2.75D model with buildings modeled as solids (Figure 2d). The boundary surfaces of the solid can be modeled using TINs (triangles) or polygons.

The above mentioned surface representations provide the geometrical model of a terrain and do not include explicit representation of individual terrain features (natural or man‐made) such as land use, buildings, roads, and water bodies. A representation of terrain features is required to support semantic queries about these features. To identify these individual terrain features one must define them as discrete objects and provide their characteristics and relations to other features explicitly through semantics. In an object perspective, a terrain can be viewed as a container populated by these objects, each with identity, spatial embedding, and attributes (Cova & Goodchild, 2002). We see here that conceptually, field and object‐based models are not mutually exclusive in case of terrains. Therefore, we describe a terrain as a:

“Continuous surface with elevation value(s) (can be more than one in case of 2.75D) for every location within its spatial domain and these locations are mapped to individual terrain objects, each with its own semantic model of information.”

### 2.2 TIN representations

**Figure 4:**
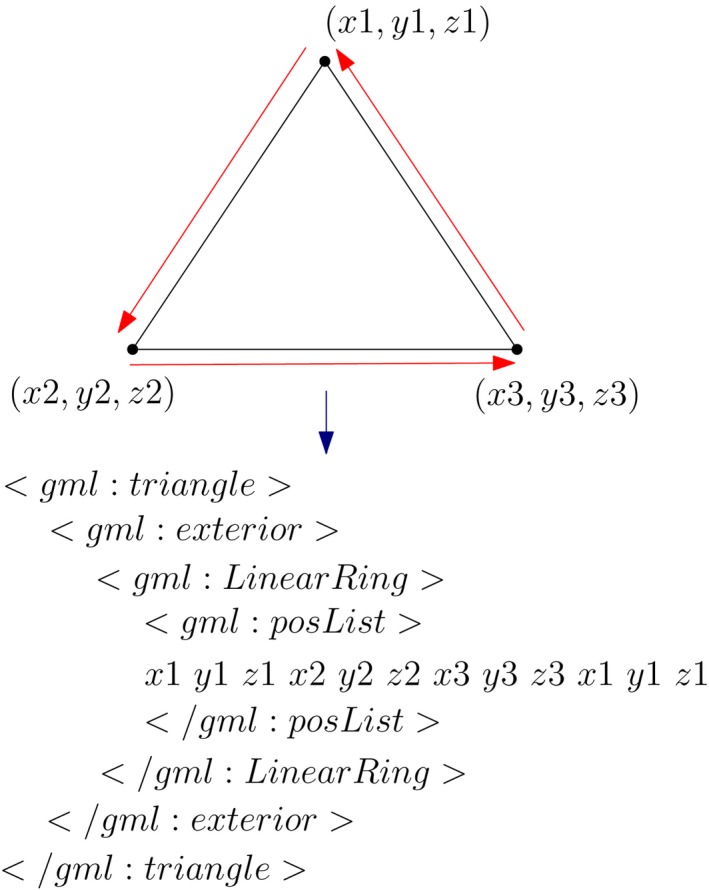
Simple Feature representation for a triangle in ISO 19136:2007 implementation standard GML (Kumar et al., 2016a). The first vertex (*x1, y1, z1*) of every triangle is repeated as the last vertex (*x1, y1, z1*) to close the linear ring

The simplest way of representing a TIN is to store each of its triangles as a list of vertex coordinates. *Simple Feature* (OGC, 2011) is an example of such a data structure. It stores each triangle as a closed linear ring of its vertex coordinates (Figure 4) (Kumar et al., 2016a). It is simple to store and represent and is supported by CityGML (GML) and almost all other spatial databases. The ISO 19136:2007 implementation standard GML uses the Simple Feature structure for storing object geometry (ISO, 2007). However, it has certain limitations. First, the structure exhibits data redundancy. In the Simple Feature structure, the first vertex of every ring is repeated as the last vertex of the linear ring (Figure 4). Given that the vertices follow a Poisson distribution, the average degree of a vertex in a 2D Delaunay triangulation is exactly 6 (Okabe, Boots, Sugihara, & Chiu, 2009). This suggests that on average each vertex is stored 6 + (6/3) = 8 times in the Simple Feature structure (Kumar et al., 2016b). The size of the dataset increases considerably with this repeated storage of vertex information for every triangle. Second, it has very limited topology and does not explicitly store the adjacency relationships between the triangles which are necessary for traversing the TIN.

The need for storage‐efficient representations for triangular meshes has contributed to the development of a number of compact data structures which have different goals, such as compression and/or explicit storage of topological relationships, for example *Indexed Triangles* (similar to OBJ), *Triangles with adjacency information *(referred to here as Triangle+) (Shewchuk, 1996; Boissonnat, Devillers, Pion, Teillaud, & Yvinec, 2002), *Stars* (Blandford, Blelloch, Cardoze, & Kadow, 2005; Ledoux, 2015), *TriStrips *(Speckmann & Snoeyink, 2001), *Half‐edge *or *DCEL* (Muller & Preparata, 1978; Mäntylä, 1987), *SQuad* (Gurung, Laney, Lindstrom, & Rossignac, 2011), *Grouper* (Luffel, Gurung, Lindstrom, & Rossignac, 2014), *Laced Ring* (Gurung, Luffel, Lindstrom, & Rossignac, 2011), *Zipper* (Gurung, Luffel, Lindstrom, & Rossignac, 2013), and *Tripod* (Snoeyink & Speckmann, 1999).

The TIN data structures that we consider in this research are *Indexed Triangles*, *Stars*, and *TriStrips*. The other data structures are also capable of reducing the storage requirements for a TIN and ensuring an efficient implementation with respect to run‐time and mesh operations. They can be useful for streaming and visualization of large TINs. CityGML, on the other hand, is an XML‐based data model for storing and representing 3D city objects. Visualization of data is not the main task of CityGML. Storing data in XML format with highly compressed data structures would require more preprocessing and later extensive decoding for comprehensibility. Therefore, we only consider simple solutions that fit in the CityGML (GML) model and still assure interoperability. We present in the following subsections the details of the data structures, and we use them for tests in Section 4.

#### 2.2.1 Indexed Triangle

**Figure 5:**
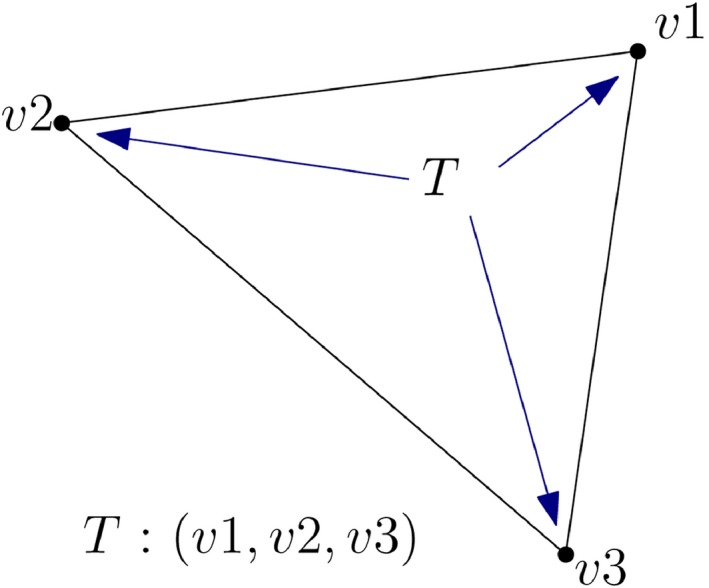
Indexed Triangle (Kumar et al., 2016b). Every triangle *T* is represented by the IDs of the three vertices $\left(v_{1},\;v_{2},\;v_{3}\right)$ forming the triangle

This stores every triangle of the TIN as references to the IDs of the three vertices forming the triangle (Kumar et al., 2016b). The vertices are stored in a separate list with IDs and are not repeated for every triangle like in Simple Feature. For instance, in Figure 5, a triangle *T* has three vertices with IDs $\left\{v_{1},\;v_{2},\;v_{3}\right\}$ each with a tuple of location coordinates (*x*, *y*, *z*). With Indexed Triangle, the triangle *T* and its vertices are represented as shown below:

**Table:** 

# list of vertices v1 = (x1,y1,z1) v2 = (x2,y2,z2) v3 = (x3,y3,z3) # list of triangles T = {v_1,v_2,v_3}

3D data formats like OBJ and ITF (Intermediate TIN Format) (VTP, 2012) use this data structure for storing triangles. The information about the adjacency and incidence relationships between the triangles of a TIN can easily be derived using this data structure.

**Figure 6:**
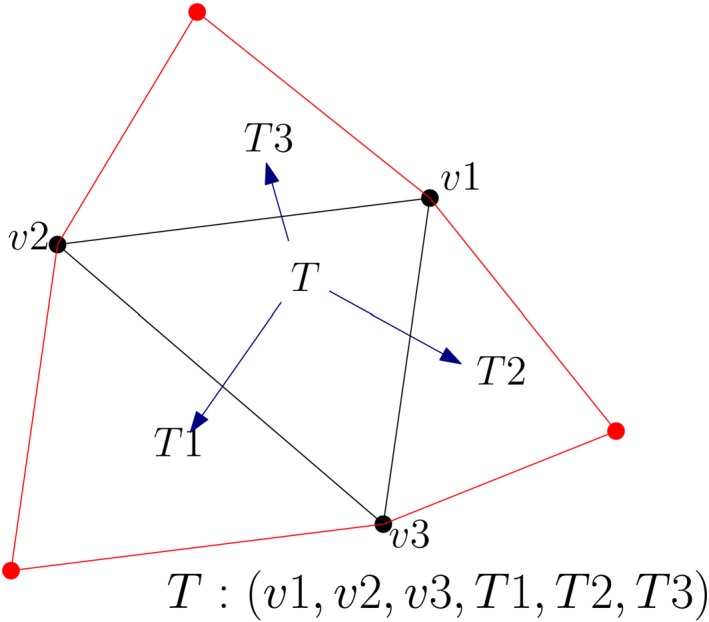
Triangle+ (Kumar et al., 2016b). Every triangle *T* is represented by the IDs of the three vertices (*v1,v2,v3*) forming the triangle and its three adjacent triangles (*T1,T2,T3*)

Another variation of the Indexed Triangle structure is Triangle+, which stores triangles along with their adjacency information. CGAL (Computational Geometry Algorithms Library) 2D triangulations (Boissonnat et al., 2002) and Shewchuk’s Triangle (Shewchuk, 1996) use this data structure. The vertex coordinates (*x*, *y*, *z*) are stored in a separate list with their IDs. Apart from storing references to the three bounding vertices (*v* ^1^,*v* ^2^,*v* ^3^,*v* ^4^,*v* ^5^,*v* ^6^), it also stores references to the three adjacent triangles {T1,T2,T3} for storing the topology (Figure 6). However, the storage requirements are increased with the presence of adjacency relationships.

#### 2.2.2 TriStrip

**Figure 7:**
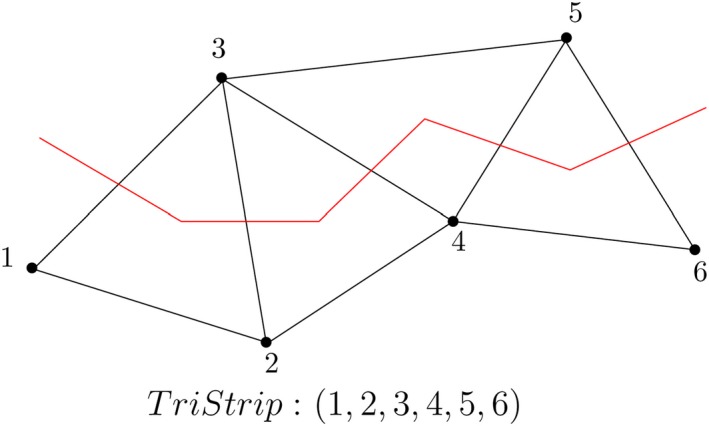
TriStrip (Speckmann & Snoeyink, 2001). The first triangle (Δ123) is formed by the first three vertices and the next triangle (Δ234) is formed by dropping the first vertex and taking up the next vertex in sequence

A TriStrip or a triangle strip is a sequence of *n* + 2 vertices that represents *n* triangles of a triangulation (Figure 7) (Speckmann & Snoeyink, 2001). TriStrips are based on the same concept as Indexed Triangles but are potentially capable of reducing the storage by a factor of 3 (Speckmann & Snoeyink, 2001). The vertex coordinates (*x*, *y*, *z*) are stored in a separate list with their IDs. To generate a TriStrip, we start with the three vertices of a triangle, then add a new vertex, and drop the oldest vertex to form the next triangle in sequence (Speckmann & Snoeyink, 2001). For instance, in Figure 7, the TriStrip (1,2,3,4,5,6) represents four triangles: Δ123 (formed by the first three vertices), Δ234 (formed by dropping the first vertex and taking up the next vertex in sequence), Δ345, and Δ456. OpenGL and 3D data standards like COLLADA support TriStrips for representing the geometry of objects.

#### 2.2.3 Star

**Figure 8:**
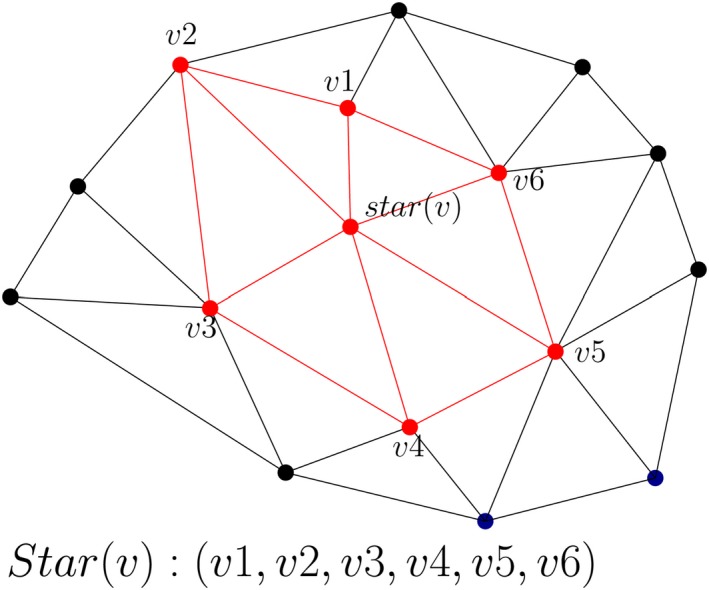
Star (Kumar et al., 2016b). Every triangle incident to the vertex *v* is represented by *v* and the two consecutive vertices in the list *v_i_* (e.g. Δvv_1_v_2_ is given by $\left\{v,\;v_{1},\;v_{2}\right\}$)

This is a vertex‐based, compressed, and pointerless data structure for compactly representing triangular meshes (Blandford et al., 2005). The star of a vertex is represented as an ordered list (counter‐clockwise) of IDs of the vertices incident on it (Ledoux, 2015); for example, in Figure 8, the star of vertex *v*, *star*(*v*), is represented by the vertex list $\left\{v_{1},\;v_{2},\;v_{3},\;v_{4},\;v_{5},\;v_{6}\right\}$. The vertex coordinates (*x*, *y*, *z*) are stored in a separate list with their IDs. The triangles are not stored explicitly but computed on‐the‐fly. Every triangle incident to the vertex *v* is represented by *v* and the two consecutive vertices in the list *v_i_* (e.g. Δvv_1_v_2_ is given by (*v*,*v* ^1^,*v* ^2^)).

### 2.3 Storing terrains in CityGML and associated problems

The data model of CityGML consists of a core module and several thematic modules for describing urban features such as Building, Relief, LandUse, Transportation, Vegetation, and WaterBody (OGC, 2012). In CityGML, terrains are defined within the thematic module Relief and represented by the class ReliefFeature in LODs 0–4 (OGC, 2012). With ReliefFeature, a terrain can be represented either as a TIN (TINReflief), or as a grid (RasterRelief), or as mass points (MasspointRelief), or as break lines (BreaklineRelief). It is also possible to represent a terrain as a combination of different terrain types in one CityGML dataset (e.g. as a TIN with break lines or as a coarse grid with some areas as TINs).

The CityGML class that we are interested in is TINReflief. It represents terrains as TINs using either gml:TriangulatedSurface or gml:Tin (GML3 geometry types). With gml:TriangulatedSurface, the triangles of a TIN are explicitly specified with Simple Feature geometry (gml:Triangle), whereas in gml:Tin, a list of 3D control points is specified along with the triangles. The support for triangles (gml:Triangle) in GML3 as Linear Rings is in accordance with the ISO 19107:2003 Spatial Schema and OGC Simple Feature Common Architecture (OGC, 2011). However, the shortcomings of the Simple Feature structure (described in Section 2.2) are clearly visible in the CityGML implementation when working with massive terrain datasets.

With advancements in 3D data acquisition and processing technologies, it is now possible to generate billions of 3D points even for an area of a few square kilometers, and, therefore, the TIN generated from these points is also massive in size. Based on the literature review and experiments conducted, we found that there are several problems in storing these massive TINs with CityGML (Kumar et al., 2016b). First, CityGML datasets become very large with the repeated storage of vertex information in the Simple Feature data structure. Second, there is very little topological information stored with Simple Feature. Each triangle is stored individually regardless of its neighbors, which hinders spatial analysis greatly. Third, there is no referencing scheme for the vertices of a triangle in the Simple Feature structure. Each of the triangles is specified with repetition of full vertex coordinate values, which takes a lot of storage space (Figure 4) (Kumar et al., 2016a). This is one of the main reasons for the increased size of CityGML datasets.

Another problem concerns the representation of vertical triangles in a TIN model. CityGML is implemented as an application schema of GML3 (OGC, 2012). The gml:Tin is based on the ISO 19107:2003 specification of GM_TIN, which in theory is a 2.5D+ structure and can have vertical triangles. However, there is no procedure in CityGML/GML to explicitly handle these vertical triangles.

## 3 Modeling a CityGML extension for massive TINs

### 3.1 CityGML extension modeling

Depending upon the application requirements, users may want to model objects and attributes of 3D city models which are not covered in the data model of CityGML. For instance, CityGML does not contain explicit thematic models for embankments, excavations, and city walls (OGC, 2012). One solution can be to model these objects using the CityGML module *Generics*. Generics is a semi‐structured extension mechanism where the city objects are extended with additional objects and attributes without making any changes in the CityGML schema. But using Generics has certain limitations. CityGML datasets with generic objects and/or attributes cannot be validated against the schema because their names and data types are not formally defined in the schema. Moreover, name conflicts of the generic attributes and objects may occur. Consequently, using Generics has very limited semantic and syntactic interoperability.

The second approach that CityGML uses to specify extensions to the model is ADE. While Generics are created at run‐time without introducing any changes in the CityGML schema, an ADE is formally specified in a separate XSD (XML Schema Definition) file and has its own namespace (OGC, 2012). ADEs are actively used by information communities to create application‐specific extensions such as the Energy ADE for energy modeling (Nouvel et al., 2015), the GeoBIM ADE for BIM‐IFC integration with CityGML (de Laat & Van Berlo, 2011), the IMGeo ADE for modeling Dutch topographic data in CityGML (Brink, Stoter, & Zlatanova, 2013), and the Noise ADE for noise modeling (OGC, 2012). The advantage of using ADEs is that the extensions are formally specified, which ensures semantic and syntactic interoperability for the exchange of application‐specific information. The extended CityGML instances can be validated. Additionally, it is possible to use more than one ADE in the same dataset.

After comparing the two alternatives, we adopted the ADE approach for modeling an extension to CityGML. ADEs can be modeled in two ways: first, directly in the XSD schema file; second, by extending the UML model of CityGML with application‐specific attributes/objects and later generating the XML schema from the UML model. Brink et al. (2013) describe six alternatives for modeling ADEs in CityGML. One approach is to add new application‐specific attributes directly in the existing CityGML classes. However, this implies editing the standard CityGML schema, which is controlled by a different authority: OGC (Open Geospatial Consortium). Alternatively, we can use ADE hooks; every CityGML feature type has a “hook” *_GenericApplicationPropertyOf<Featuretypename>* in its XML schema definition which allows attaching an arbitrary number of additional attributes to it in the ADEs. Another approach is to add new attributes or objects in subclasses in an ADE package. Since we are modeling an extension to CityGML, defining the new classes as subclasses of existing CityGML classes and adding the new attributes to these subclasses seems appropriate. Therefore, we prefer to adopt this approach for modeling the ADE. The method of inheritance with classes and subclasses is easy to understand with some basic knowledge of UML. This approach was also accepted as best practice by OGC (2014).

### 3.2 Modeling choices for new TIN geometry types in GML

**Figure 9:**
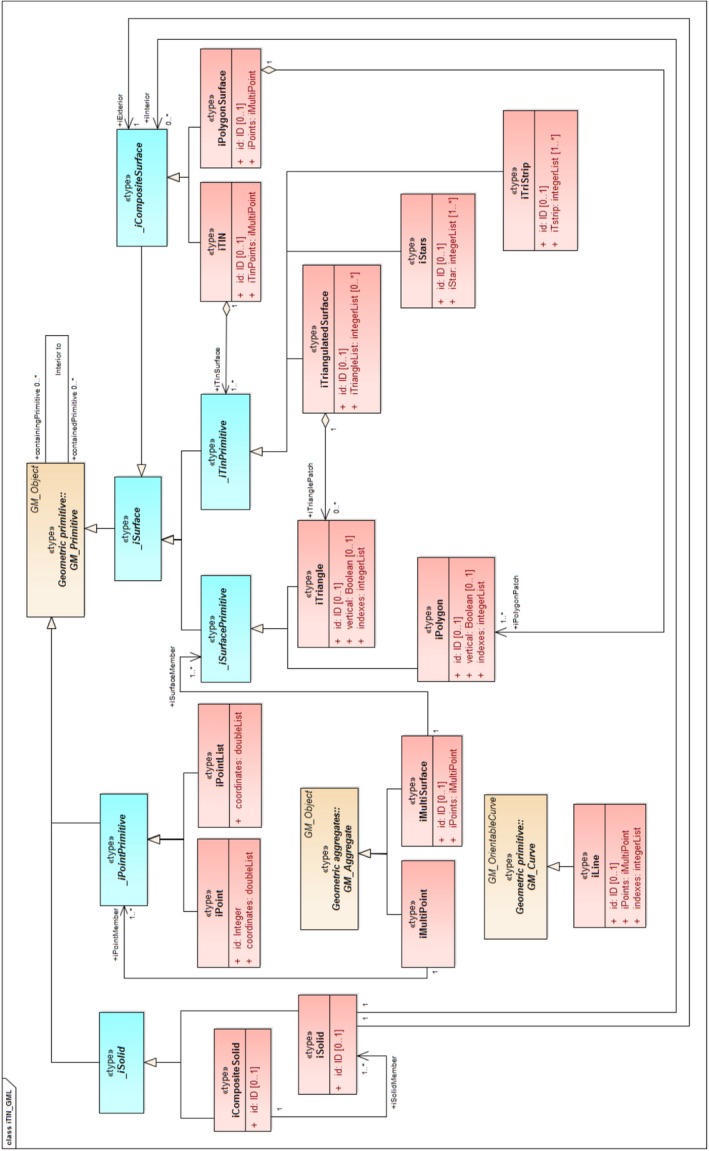
Our proposed geometry types in the GML3 geometric model for massive TINs (proposed abstract classes are shown in blue and implementation classes are shown in red)

CityGML features are spatially represented by the GML3 geometry model. The geometry model of GML3 is based on the ISO 19107:2003 “Spatial Schema” (ISO, 2003). It consists of geometric primitives such as points, lines, and polygons, which are combined to form complexes, aggregates, or composite geometries. Therefore, we introduce the new geometry types in the GML3 geometry model (see Figure 9) and extend them to CityGML feature types as an ADE.

To avoid any name conflict with the existing GML elements, the new schema elements are defined in a separate XSD file iTIN_GML.xsd with a different namespace "https://godzilla.bk.tudelft.nl/schemas/iTIN_GML" and the igml identifier. We introduce new geometry types (primitives, aggregates, and composites) in this model for compactly representing TINs (see Table 1). New abstract classes for representing these geometry types are added so as not to disturb the original hierarchy of the GML3 model.

- igml:_iPointPrimitive. An *_iPointPrimitive* is an abstract class for modeling the point geometries. It is modeled as a type of *gml:_GeometricPrimitive*.
- igml:iPoint. An *iPoint* (or *indexed Point*) represents the geometry of an individual point (or vertex). It is modeled as a type of *igml:_iPointPrimitive* *.* Each iPoint has an integer ID and a list of its coordinates (*x*, *y*, *z*) given by <igml:id> and <igml:coordinates>, respectively. An igml:iPoint representation for a point is given below:

**Table 1:** Proposed iTIN_GML geometry elements. Prefix “*i*” signifies that everything is indexed and refers to the extension we proposed to the model. Prefix “_” indicates an abstract class in the model

#	iTIN_GML	Base class	
		GML	iTIN_GML
1.	*_iPointPrimitive*	*_GeometricPrimitive*	
2.	iPoint		*_iPointPrimitive*
3.	iPointList		*_iPointPrimitive*
4.	iMultiPoint	*_GeometricAggregate*	
5.	iMultiSurface	*_GeometricAggregate*	
6.	iLine	*_Curve*	
7.	*_iSurface*	*_Surface*	
8.	*_iSurfacePrimitive*		*_iSurface*
9.	iTriangle		*_iSurfacePrimitive*
10.	iPolygon		*_iSurfacePrimitive*
11.	*_iTinPrimitive*		*_iSurface*
12.	iTriangulatedSurface		*_iTinPrimitive*
13.	iTriStrip		*_iTinPrimitive*
14.	iStars		*_iTinPrimitive*
15.	*_iCompositeSurface*		*_iSurface*
16.	iTIN		*_iCompositeSurface*
17.	iPolygonSurface		*_iCompositeSurface*
18.	*_iSolid*	*_GeometricPrimitive*	
19.	iSolid		*_iSolid*
20.	iCompositeSolid		*_iSolid*

**Table:** 

<igml:iPoint> <igml:id>1234</igml:id> <igml:coordinates> 85027.492 447446.125 1.51 </igml:coordinates> </igml:iPoint>

- igml:iPointList. An *iPointList* (or *indexed Point List*) is a list of all the points (or vertices) of a surface defined by space‐separated values of all the coordinates. It is modeled as a type of *igml:_iPointPrimitive*.

**Table:** 

<igml:iPointList> <igml:coordinates> 85027.492 447446.125 1.51 85027.289 447446.156 1.31 85049.219 447448.312 1.37 85068.219 447447.332 1.64 .... <igml:coordinates> </igml:iPointList>

- igml:iMultiPoint. To represent all the points of a surface, we added a new class igml:iMultiPoint. An *iMultiPoint* is a collection of all the points (i.e. vertices) of a surface and is a type of *gml:_GeometricAggregate* *. *With igml:iMultiPoint it is possible to store points either as a collection of individual igml:iPoint(s) referenced through igml:iPointMember elements or as a igml:iPointList (see snippet below).

**Table:** 

<igml:iMultiPoint> <igml:iMultiPoint> <igml:iPointMember> <igml:iPointMember> <igml:iPoint> <igml:iPointList> .... .... </igml:iPoint> </igml:iPointList> <igml:iPointMember> </igml:iPointMember> .... </igml:iMultiPoint> </igml:iMultiPoint>

- igml:iLine. An *iLine* (or *indexed Line*) represents the geometry of an individual line segment (or curve). It is modeled as a type of *gml:_Curve* which is a subtype of *gml:_GeometricPrimitive* *.* We did not introduce any separate abstract base class (such as *_iLine*) because it is a complete geometry (with points and indexes) and hence can be reused with gml:MultiCurve. The existing hierarchy of elements in the GML model is followed for defining new classes in the model. Each iLine has an ID given by <igml:id> and a list of IDs of the points forming the line given by <igml:indexes>. The <igml:indexes> lists the IDs of the points comprising the geometry instead of repeating the coordinate values of the points again. An igml:iLine representation for a line connecting two points (with given point IDs) is defined as:

**Table:** 

<igml:iLine> <igml:id>D23</igml:id> <igml:iPoints> .... </igml:iPoints> <igml:indexes>1 2</igml:indexes> </igml:iLine>

- *igml:_iSurface* *.* For modeling the surfaces, we introduced another abstract class *igml:_iSurface* as a type of *gml:_GeometricPrimitive*. It has three subclasses: igml:_iSurfacePrimitive for modeling individual surface elements (polygon and triangle), *igml:_iTinPrimitive* for modeling TIN representations, and *igml:_iCompositeSurface* for modeling TINs and composite polygonal surfaces.
- igml:iTriangle. An *iTriangle* (or *indexed Triangle*) represents the geometry of an individual triangle. It is modeled as a type of *igml:_iSurfacePrimitive* *.* An igml:iTriangle is specified by the references to IDs of the three vertices of the triangle given by gml:iPoint. It has an optional element igml:vertical to specify if the triangle is a vertical triangle. For some applications such as flow modeling, adjacency, and network analysis, it is sufficient to use a city model and its buildings as a single triangulated surface containing vertical triangles instead of using a volumetric model (Gorte & Lesparre, 2012). The <igml:vertical> element helps us to identify these vertical surfaces modeled in the terrain without relying on the geometry and on‐the‐fly computation (which are prone to precision errors). This means that the model is more than 2.5D but less than 3D; the geometry is 3D, but the underlying topology remains 2D.

**Table:** 

<igml:iTriangle> <igml:id>34</igml:id> <igml:vertical>false</igml:vertical> <igml:indexes>1 2 3</igml:indexes> </igml:iTriangle>

- igml:iPolygon. An *iPolygon* (or *indexed Polygon*) represents the geometry of an individual polygon. It is also modeled as a type of *igml:_iSurfacePrimitive* and has the same geometrical representation as igml:iTriangle. An igml:iPolygon is specified by the references to IDs of the vertices (>3) of the polygon. It also has an optional element igml:vertical to specify if the polygon is a vertical surface.

**Table:** 

<igml:iPolygon> <igml:id>14</igml:id> <igml:vertical>true</igml:vertical> <igml:indexes>3 4 5 6</igml:indexes> </igml:iTriangle>

- igml:iMultiSurface. An *iMultiSurface* is a collection of surfaces (triangles/polygons) which can be disjoint, overlapping, touching, or even disconnected. It is modeled as a type of *gml:_GeometricAggregate* *.* We did not introduce any separate abstract base class (such as *_iGeometricAggregate*) because it is a complete geometry (with points and indexes) and hence can be reused in other geometry types. With igml:iMultiSurface it is possible to store a surface either as a collection of triangles (igml:iTriangle) or as a collection of polygons (igml:iPolygon) referenced through igml:iSurfaceMember elements (see snippet below).

**Table:** 

<igml:iMultiSurface> <igml:iMultiSurface> <igml:id>f24</igml:id> <igml:id>f24</igml:id> <igml:iSurfaceMember> <igml:iSurfaceMember> <igml:iTriangle> <igml:iPolygon> .... .... </igml:iTriangle> </igml:iPolygon> </igml:iSurfaceMember> </igml:iSurfaceMember> .... .... </igml:iMultiSurface> </igml:iMultiSurface>

- igml:_iCompositeSurface. To model disjoint, non‐overlapping, topologically connected surfaces, we introduced an abstract base class *igml:_iCompositeSurface*. It has two subclasses igml:iTIN and igml:iPolygonSurface.
- igml:iTIN. For representing TINs, we added a new class igml:iTIN as a subclass of *igml:_iCompositeSurface* (and not aggregates) because TINs represent surfaces with disjoint, non‐overlapping, and topologically connected triangles. Apart from the above mentioned geometric primitives and aggregates, we added three new TIN representation types: igml:iTriangulatedSurface, igml:iStars, and igml:iTriStrips as subclasses of *igml:_TinPrimitives*. In igml:iTIN the TIN vertices are represented using igml:iMultiPoint and the TIN surface can be represented using any of these three new surface types.

**Table:** 

<igml:iTIN> <igml:id>A24</igml:id> <igml:iTinPoints> <igml:iMultiPoint> .... </igml:iMultiPoint> </igml:iTinPoints> <igml:iTinSurface> ..... <igml:iTinSurface> </gml:iTIN>

- igml:iPolygonSurface. For representing topologically connected polygon surfaces, we added a new class igml:iPolygonSurface as a subclass of *igml:_iCompositeSurface* *.* Points are represented using igml:iMultiPoint and the polygons are represented using igml:iPolygon geometry referenced through igml:iPolygonPatch elements.

**Table:** 

<igml:iPolygonSurface> <igml:id>A22</igml:id> <igml:iPoints> .... </igml:iPoints> <igml:iPolygonPatch> <igml:iPolygon> ..... </igml:Polygon> <igml:iPolygonPatch> </gml:iPolygonSurface>

- igml:iTriangulatedSurface. An *iTriangulatedSurface* stores triangles either as a collection of individual igml:iTriangle referenced through igml:iTrianglePatch elements or as igml:iTriangleList (see snippet below). An igml:iTriangleList is a space‐separated list of IDs of the vertices of all the triangles.

**Table:** 

<igml:iTriangulatedSurface> <igml:iTriangulatedSurface> <igml:id>{A24}</igml:id> <igml:id>{A24}</igml:id> <igml:iTrianglePatch> <igml:iTriangleList> <igml:iTriangle> 1 2 3 2 3 4 3 4 5.. .... .... </igml:iTriangle> </igml:iTriangleList> </igml:iTrianglePatch> .... .... </igml:iTriangulatedSurface> </igml:iTriangulatedSurface>

- igml:iTriStrip. An *iTriStrip* is a collection of triangles represented by igml:iTStrip elements. In each *iTstrip* the first triangle is formed from first, second, and third vertices. Each subsequent triangle is formed from the next vertex in sequence, reusing the previous two vertices. Each igml:iTriStrip can have any number of igml:iTstrip elements to depict local connectivity.

**Table:** 

<igml:iTriStrip> <igml:id>B54</igml:id> <igml:iTstrip id = "1"> 1 2 3 4 5 </igml:iTstrip> <igml:iTstrip id = "2"> 11 12 13 14 </igml:iTstrip> .... </igml:iTriStrip>

- igml:iStars. An *iStars* is a collection of igml:iStar elements defined for every vertex of a triangulated surface. For every vertex, an *iStar* *s* stores an ordered list of IDs of the vertices incident to it (see snippet below). Every triangle incident to a vertex is represented by the ID of that vertex and the IDs of two consecutive vertices in the list.

**Table:** 

<igml:iStars> <igml:id>A34</igml:id> <igml:iStar id = "1">2 3 4 5 6 7</igml:iStar> <igml:iStar id = "2">3 4 7 8 9 11</igml:iStar> .... </igml:iStars>

- igml:_iSolid. For representing solids, we introduced another abstract class *igml:_iSolid* *.* It is modeled as a type of *gml:_GeometricPrimitive* *.*
- igml:iSolid. This is modeled as a type of *igml:_iSolid* with the exterior and interior of the solid modeled as a composite surface *igml:_iCompositeSurface*. The exterior shell and interior of the solid can be modeled either as a TIN (igml:iTIN) or as a polygonal surface (igml:iPolygonSurface) referenced through igml:iExterior and igml:iInterior elements.

**Table:** 

<igml:iSolid> <igml:id>A234</igml:id> <igml:iExterior> <igml:iTIN> ..... </igml:iTIN> </igml:iExterior> <igml:iInterior> <igml:gml:iPolygonSurface> ..... </igml:gml:iPolygonSurface> </igml:iInterior> </igml:iSolid>

- igml:iCompositeSolid. This is modeled as a type of *igml:_iSolid* *. *It is a collection of solids (igml:iSolid) referenced through igml:iSolidMember elements.

**Table:** 

<igml:iCompositeSolid> <igml:id>A1234</igml:id> <igml:iSolidMember> <igml:iSolid> ..... </igml:iSolid> </igml:iSolidMember> ..... </igml:iCompositeSolid>

### 3.3 Extending CityGML for massive terrains

For modeling terrains as TINs, the iTIN_GML elements are added to CityGML using an ADE. The initial idea was to integrate these TIN representations directly in the GML model so as to use the same namspace and identifier of GML. CityGML would then inherit these geometry types automatically from the enhanced GML model. This would have eliminated the need to extend the existing CityGML classes with these new geometrical representations. However, both GML and CityGML are controlled by a formal authority: OGC. It would have been unwise to change the original GML and CityGML model without the approval of the OGC.

Therefore, to show the benefits of this approach, we developed it as an ADE. We created a separate package to model the new TIN geometry types and added them to CityGML by extending the existing CityGML classes in an ADE package. Moreover, these geometry types can easily be added to the original GML/CityGML model, if approved by the OGC.

**Figure 10:**
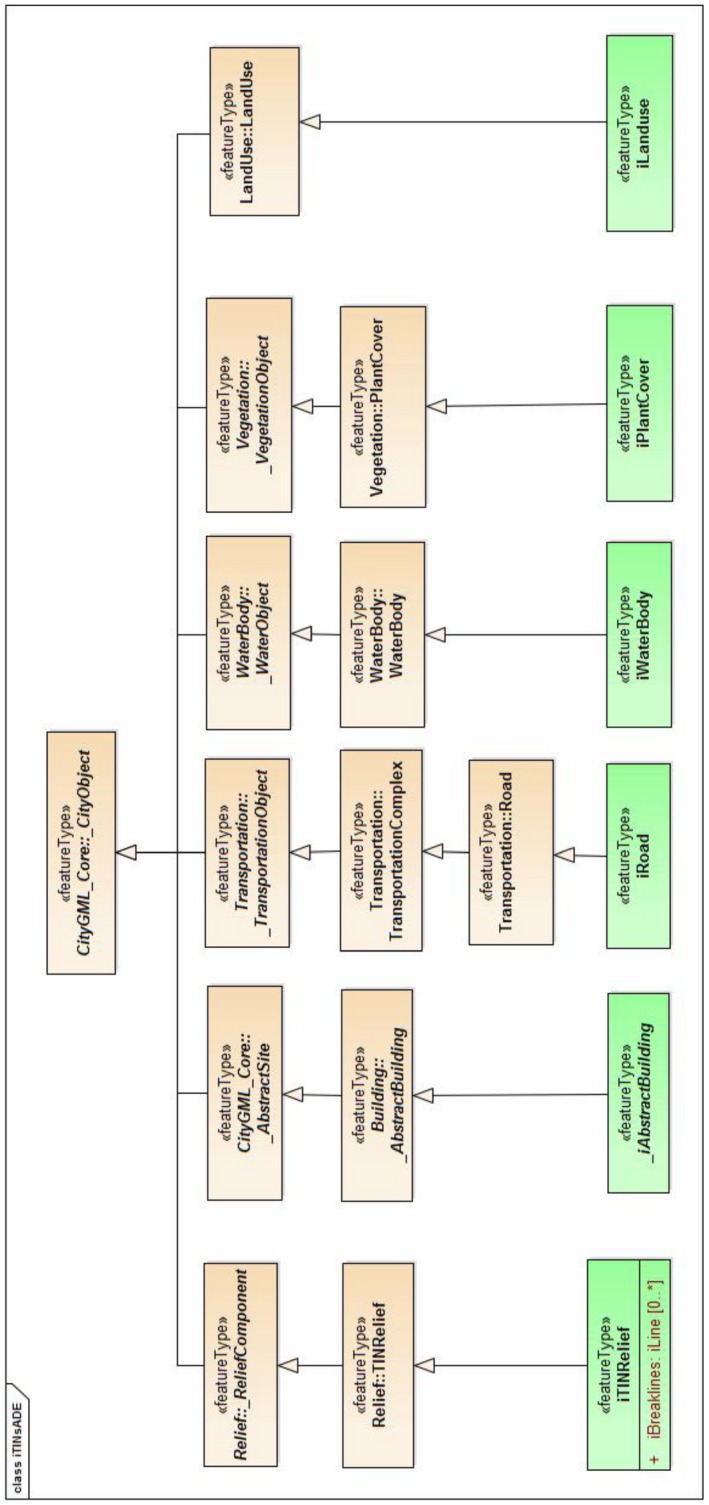
Proposed classes in CityGML iTINs ADE for massive terrains (ADE classes shown in green)

The new classes are modeled as subclasses of the existing CityGML classes (marked with stereotype <<featureType>>) and can have their own properties (Table 2). The CityGML Relief module is extended to include the iTIN_GML elements for modeling terrains (see Figure 10). Similarly, we extended other CityGML modules, Relief, Building, Vegetation, Transportation(Road), WaterBody, and LandUse to include the iTIN_GML elements for representing TINs. These elements can be used independently for compact geometrical representation of terrain and its features such as buildings, roads, and vegetation. The ADE classes are defined in a separate file CityGML_iTINs_ADE.xsd with a different namespace "https://godzilla.bk.tudelft.nl/schemas/iTINs_ADE" and the itin identifier.

**Figure 11:**
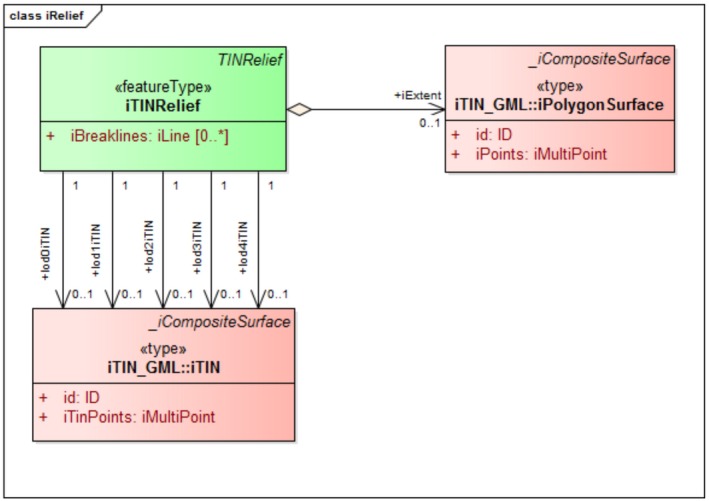
iTINRelief modeled in CityGML iTINs ADE using iTIN_GML

- iTINRelief. In the CityGML Relief module, a new relief component called iTINRelief is introduced as a subclass dem:TINRelief. iTINRelief extends all the properties of the base class like name, description, and LOD, and has igml:iTIN geometrical representation (Figure 11). In the original dem:TINRelief class, the LOD is specified using dem:lod element. Here, we introduced separate geometrical representations for the relief LODs (0–4) using lod0iTIN, lod1iTIN, lod2iTIN, lod3iTIN, and lod4iTIN elements. Another element called iExtent is also introduced to mark the extent of the TIN using igml:iPolygonSurface geometry. To represent the break lines in a TIN, we introduced an element called iBreaklines with geometry igml:iLine.

**Table 2:** New classes in the CityGML ADE (iPS = iPolygonSurface, iMS = iMultiSurface)

#	CityGML iTINs ADE	CityGML module	iTIN_GML geometry			
			iTIN	iPS	iMS	iSolid
1	iTINRelief	Relief	*✓*			
2	iWaterBody	WaterBody	*✓*	*✓*	*✓*	
3	iRoad	Transportation	*✓*	*✓*	*✓*	
4	iPlantCover	Vegetation	*✓*	*✓*	*✓*	
5	iLandUse	LandUse	*✓*	*✓*	*✓*	
6	_iAbstractBuilding	Building	*✓*	*✓*		*✓*

**Table:** 

<cityObjectMember> <dem:ReliefFeature> <gml:name> Example iTINRelief </gml:name> <dem:lod> 1 </dem:lod> <dem:reliefComponent> <itin:iTINRelief> <dem:lod> 1 </dem:lod> <itin:iTINobject> <itin:lod1iTIN> <igml:iTIN> ... </igml:iTIN> <itin:lod1iTIN> </itin:iTINobject> </itin:iTINRelief> </dem:reliefComponent> </dem:ReliefFeature> </cityObjectMember>

**Figure 12:**
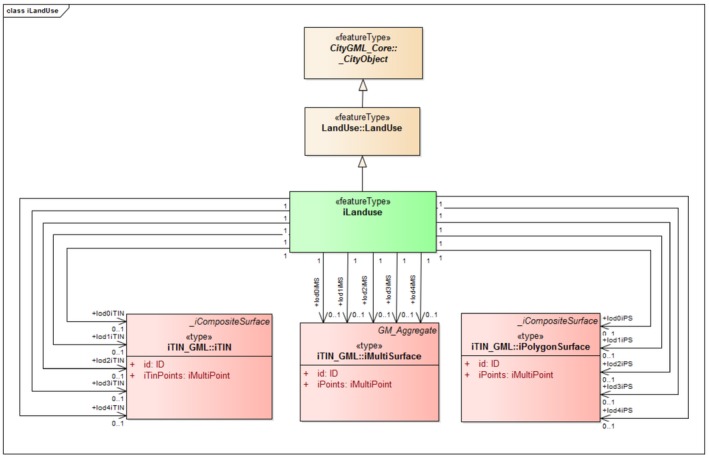
iLandUse modeled in CityGML iTINs ADE using iTIN_GML

**Figure 13:**
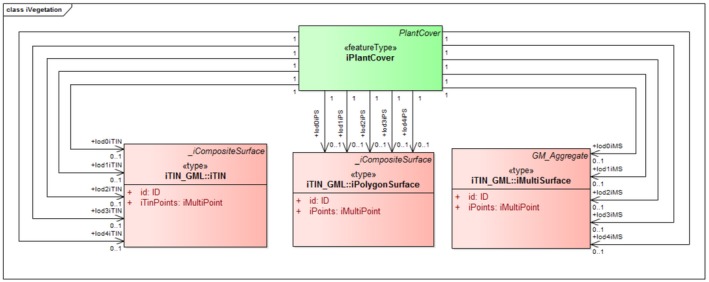
iPlantCover modeled in CityGML iTINs ADE using iTIN_GML

**Figure 14:**
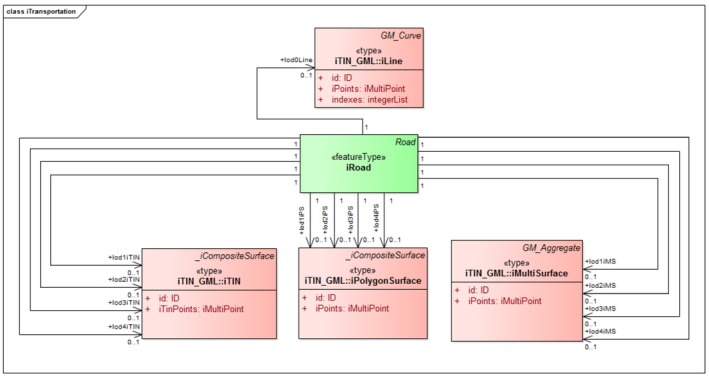
iRoad modeled in CityGML iTINs ADE using iTIN_GML

**Figure 15:**
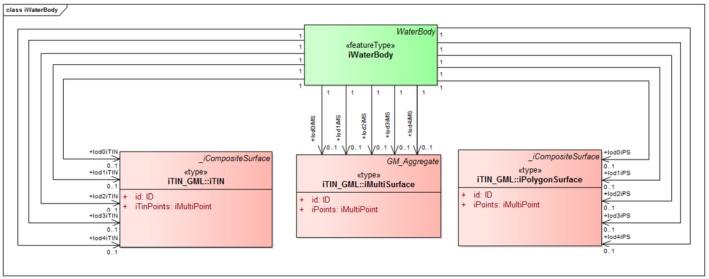
iWaterBody modeled in CityGML iTINs ADE using iTIN_GML

**Figure 16:**
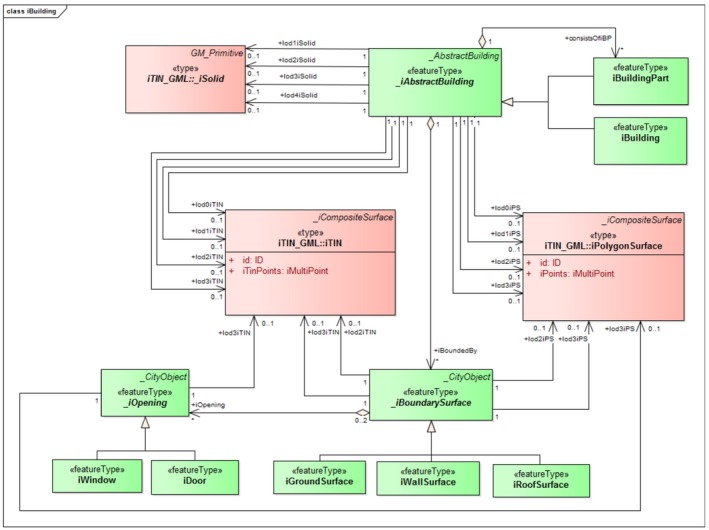
*_iAbstractBuilding* modeled in CityGML iTINs ADE using iTIN_GML

- iLandUse. In the CityGML LandUse module, a new component called iLandUse is introduced as a subclass luse:LandUse. iLandUse extends all the properties of the base class like name, description, and LOD. It can be represented either with igml:iTIN, or igml:iPolygonSurface, or igml:iMultiSurface geometrical representations at different LODs (0–4) (Figure 12).
- iPlantCover. In the CityGML Vegetation module, a new component called iPlantCover is introduced as a subclass veg:PlantCover. The Vegetation module has class veg:SolitaryVegetationObject to model single vegetation objects, and class veg:PlantCover to model areas filled with a specific vegetation type. Solitary vegetation objects are usually modeled with implicit geometries, therefore we added iTIN_GML representations only to veg:PlantCover. Vegetation can be represented with iPlantCover using either igml:iTIN, or igml:iPolygonSurface, or igml:iMultiSurface geometrical representations at different LODs (0–4) (Figure 13).
- iRoad. In the CityGML Transportation module, a new component called iRoads is introduced as a subclass tran:Roads. The road is represented as a tran:TransportationComplex in CityGML with different geometrical representation at different levels of detail. At LOD 0, iRoads use igml:iLine geometry for representing roads. For LODs 2–4, iRoads can be represented using either igml:iTIN, or igml:iPolygonSurface, or igml:iMultiSurface geometrical representations (Figure 14). In CityGML, objects such as *Track*, *Road*, *Railway*, and *Square* are also modeled as a type of tran:TransportationComplex. These objects are beyond the scope of this study and, therefore, are not included in the ADE. However, we assure that these objects can be extended in a similar manner for representation.
- iWaterBody. In the CityGML WaterBody module, a new component called iWaterBody is introduced as a subclass of wtr:WaterBody. Water can be represented with iWaterBody using either igml:iTIN, or igml:iPolygonSurface, or igml:iMultiSurface geometrical representations at different LODs (0–4) (Figure 15). Theoretically, in CityGML, any WaterBody can also be represented by a solid, bounded by thematic surfaces, at LODs 2–4 (OGC, 2012). In real‐world scenarios it is usually modeled as a surface and therefore we do not take solid representation into account. However, it can be added to the ADE in the same way as surface representation.
- _iAbstractBuilding. In the CityGML Building module, a new abstract class *_iAbstractBuilding* is added as a subclass of *bldg:_AbstractBuilding* *.* *_iAsbtractBuilding* has two subclasses: iBuilding and iBuildingPart. Buildings and building parts can be represented either with igml:iSolid, or igml:iTIN, or igml:iPolygonSurface geometric representation (Figure 16). *_iAsbtractBuilding* is modeled for LODs 0–3. Openings and boundary surfaces are also represented for modeling LOD 3. LOD 4 with building interior can be modeled in the same manner.

## 4 Implementation and experiments with real‐world datasets

### 4.1 *iTIN_GML* *and CityGML iTINs ADE schema generation*

We used the ShapeChange (https://shapechange.net) tool to derive the XML schemas of the iTIN_GML and CityGML iTINs ADE from the UML model. ShapeChange is a Java‐based tool which implements UML to GML encoding rules described in ISO 19136, ISO 19118, and ISO 19109. We only generated the XML schema for the GML and CityGML extensions and not for the whole data models as they are already publicly available. The generated CityGML ADE schema only requires importing the existing CityGML schema and the generated iTIN_GML schema containing new geometry types for representing TINs. These dependencies are resolved by ShapeChange during the transformation from UML packages to XML schema. The UML models and XML schemas for iTIN_GML and CityGML iTINs ADE are available at https://github.com/tudelft3d/CityGML_iTINs_ADE.

### 4.2 Prototype testing

The terrain datasets used for testing the implementation are as follows.

1. *AHN3 TIN*. AHN3 (Actueel Hoogtebestand Nederland version 3) (AHN2015, 2015) is the national height model of the Netherlands and contains billions of 3D points (more than 10 points/m^2^). AHN3 Tile# 37EN/1 (size 5 × 6.25 km^2^) of the AHN3 point cloud is used as input for generating the TIN using Lastools (Hug, Krzystek, & Fuchs, 2004). The dataset is generated in streaming mesh format (*.sma) (Isenburg, Lindstrom, Gumhold, & Snoeyink, 2005).
2. *3DBGT*. 3DBGT (3D Basisregistratie Grootschalige Topografie) is the 3D city model of the Netherlands created using the open‐source software *3dfier* (https://3d.bk.tudelft.nl/opendata/3dfier/). 3DBGT is a constrained triangulation generated from AHN3 point cloud and 2D BGT (large‐scale 2D topographic dataset of the Netherlands) footprints (BGT, 2016). 3dfier takes 2D topographic datasets and lifts every 2D polygon to the required height to make them 3D. This height information is obtained from the point cloud data. We used 3DBGT TIN of the Amsterdam area for testing. The dataset is available in OBJ format (*.obj).
3. *3DTOP10NL*. 3DTOP10NL is the 3D city model of the Netherlands, which covers the whole country, including buildings, terrain, roads, canals, and so on in 1,368 tiles. It is generated by adding the height information from AHN2 point cloud to the 2D topographic objects in TOP10NL (Elberink, Stoter, Ledoux, & Commandeur, 2013). The layer that we are interested in for the 3DTOP10NL dataset is the “terreinVlak_3D_LOD0”, which contains the terrain model with more than 1 billion triangles. The dataset is available in ESRI GeoDatabase format (*.gdb).

The details of the input terrain datasets along with their size in CityGML format are given in Table 3. A prototype was created to introduce new TIN representations in CityGML datasets. The prototype reads the input datasets and maps the Simple Feature representation of triangles to the index‐based structure of igml:iTIN. The resulting storage sizes of the prototype testing are given in Table 3, along with the achieved compression factors.

**Table 3:** Details of the input datasets showing the number of triangles in each terrain dataset and the storage space required for storing each dataset in CityGML and CityGML iTINs ADE format (CF = Compression Factor, iTS = iTriangulatedSurface)

Terrain dataset	Number of triangles	CityGML file size	iTS		iTriStrip		iStars	
			size	CF	size	CF	size	CF
3DBGT	13,688,402	4.65 GB	337.92 MB	14.32	236.54 MB	20.1	816.13 MB	5.83
Tile #37EN/1	40,788,573	13.98 GB	952.32 MB	15.13	747.52 MB	19.22	2.28 GB	6.13
3DTOP10NL	1,156,641,666	698.74 GB	46.64 GB	14.38	37.43 GB	18.67	108.33 GB	6.45

We also compared the time taken to generate data in CityGML and CityGML iTINs ADE formats from original test datasets (Table 4) to observe the performance of the system in handling massive terrain data. These tests were performed on a Linux Godzilla server with 40 Intel Xeon E5‐2650 v3 CPUs, 128 GB RAM, 3.3 GHz base clock speed, and 3.6 GHz turbo boost speed. The three test datasets are available in three different formats (OBJ, SMA, and GDB) and the time taken to generate output data from these datasets differs significantly. From Table 4 we can see that it takes less time to generate CityGML data from the 3DTOP10NL GeoDatabase. This can be attributed to the fact that both CityGML and Esri GeoDatabase follow the Simple Feature structure for representing geometry. While generating iTIN_GML geometry types from this Simple Feature structure most of the time is consumed in cleaning the vertices (removing duplicates), generating integer IDs for the vertices, and assigning these indexes to the triangles for representing the geometry. However, in case of other formats like OBJ and SMA, which already follow a simple indexing scheme, the igml:iTriangulatedSurface structure is generated very quickly. For igml:iTriStrip and igml:iStars the data generation time is a bit high as it also includes the time taken to compute the neighboring triangles/vertices (required for TIN traversal).

**Table 4:** Time taken to generate CityGML and CityGML iTINs ADE data from test datasets

Terrain dataset	CityGML (min)	iTS (min)	iTriStrip (min)	iStar (min)
3DBGT (obj)	25.63	15.68	63.83	27.21
Tile #37EN/1 (sma)	52.19	38.87	93.77	54.32
3DTOP10NL (gdb)	38.54	121.63	194.31	166.91

We also tested for the storage size of quantized vertices (Isenburg, Lindstrom, & Snoeyink, 2005). A vertex is called quantized when we store only the difference of its coordinates from the centroid vertex (or any other vertex) and not the full vertex coordinates. The centroid vertex is the centroid of the vertices of the TIN or can also be selected randomly. We also tried storing the difference of the coordinates from the first vertex of the TIN. However, storing quantized vertices did not change the compression factors significantly. As this was not the main objective of our study, we did not test it further.

As can be observed from the results, the highest compression factor of 20.1 is achieved using the *iTriStrip* referencing scheme for storing TINs in place of the Simple Feature structure. The data structures in decreasing order of storage requirements are:

iStars > iTriangulatedSurface > iTriStrip

**Figure 17:**
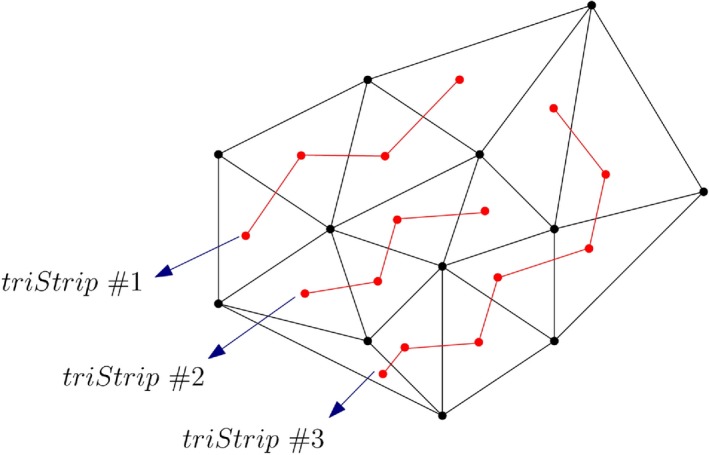
Flow diagram for generating triangle strips from the CityGML test datasets

**Figure 18:**
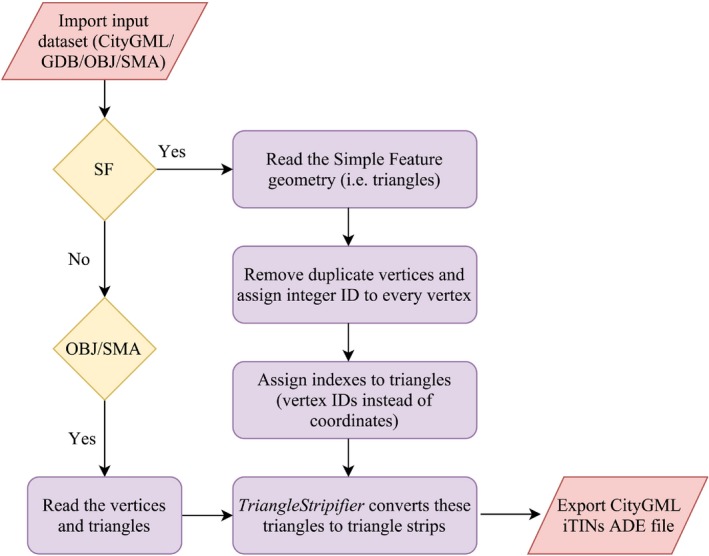
A single TIN can have a number of disconnected triangle strips. There is local connectivity within each strip (shown in red) but no overall connectivity for the entire TIN

Although the inclusion of triangle strips (*iTriStrips*) provides maximum reduction in storage size, it has certain topological restrictions. We used the *TriangleStripifier* module of the *PyFFI* python package to generate triangle strips for our datasets (PyFFI, 2011). *TriangleStripifier* is a python adaptation of the *NvTriStrip* library (NVIDIA, 2004) and converts triangles into a list of strips. A triangle strip enters each triangle at one edge (known as the *entry‐edge*) and exits that triangle on the left or the right remaining edges (known as *exit‐edges*) (Speckmann & Snoeyink, 2001). The triangle strip alternates between left and right exit‐edges with each successive triangle until it reaches a triangle with no forward connections (Speckmann & Snoeyink, 2001). For the remaining triangles, the same process is repeated until all the triangles are placed in triangle strips. The process of generating triangle strips from the test datasets is depicted in Figure 17. Therefore, for a single TIN, we can have a number of disconnected triangle strips storing the mesh triangles (Figure 17, 18). This means there is local topological connectivity within the individual triangle strips but no overall connectivity for the entire TIN. Certain operations are thus not possible in constant time, such as finding the adjacent triangles of a given triangle.

This is not the case with *Stars*. When all the stars in a TIN are represented, each triangle is present in exactly three stars (its three vertices) and each edge is present in exactly two stars (its two vertices) (Ledoux, 2015). There is a significant overlap in the stars from which we can derive the adjacency and incidence relationships of the triangles of a TIN (Ledoux, 2015). For a given vertex we can easily find the incident edges or triangles using stars. Therefore, these data structures in increasing order of topology can be represented as:

iTriStrip < iTriangulatedsurface < iStars

## 5 Conclusions and further research

This article presents a new CityGML extension for efficiently storing massive TIN terrains in CityGML. We investigated several TIN data structures for their storage requirements and topology storage, and explored how they can be implemented in CityGML for storing massive TINs. We introduced three new index‐based geometry types (*Indexed Triangles*, *TriStrips*, and *Stars*) for representing TINs in the GML schema and extended them to CityGML as an ADE. Our approach allows us to store TIN terrains in CityGML with nearly 20 times less storage than the Simple Feature structure in CityGML. This CityGML ADE addresses the issues of massive size of TIN terrain datasets, and explicit handling of vertical triangles in these datasets. It is a stepping stone in the direction of reducing the large size of CityGML datasets while still maintaining usability for different applications.

CityGML differentiates five consecutive LODs (LOD 0 to LOD 4), wherein features become much more detailed in their geometry and semantic differentiation with each increasing LOD (OGC, 2012). This LOD concept is very well established in the case of buildings, bridges, and roads; however, this is not the case with other CityGML modules like relief (terrain), land use, and vegetation (Biljecki, Ledoux, & Stoter, 2016; Löwner, Gröger, Benner, Biljecki, & Nagel, 2016). For instance, the LOD of a relief object is expressed as integer attribute gml:lod with values between 0 and 4. We added new elements *lod1iTIN*, *lod2iTIN*, …, *lod4iTIN* in the CityGML Relief (and other modules) to model different LODs of the terrain. However, the proper specification to model the geometry and semantics of terrains at each LOD is still missing in the CityGML specifications. The CityGML specifications do not distinguish between different terrain LODs at the geometric and semantic level, although it is possible to model different levels of terrain (Luebke, 2003). Since a terrain is a depiction of location–elevation values, it cannot always be an otherwise flat LOD 0 model with one elevation value per triangle in a TIN. A terrain model can also have vertical walls and overhangs. Our future plan is to extend the concept of LODs for terrains and include it in the CityGML semantic model of the ADE.

The next step is to integrate this ADE into the database to see its overall performance in handling terrain data. We plan to use 3DCityDB (https://www.3dcitydb.org/) (PostgreSQL) for the database implementation of the ADE. Our previous tests have shown that it takes a significantly larger amount of time to populate and index the TIN datasets with the Simple Feature structure than the index‐based data structures in the database (Kumar et al., 2016a, 2016b). In the case of the ADE, we expect that the loading time from the CityGML ADE file to the database will most likely improve. The spatial index will be smaller as it does not require creating a complex spatial index like giST (in case of Simple Feature). The indexing can be accomplished at the vertex level with a simple B‐tree (Ledoux, 2015; Kumar et al., 2016a, 2016b).

CityGML is designed for the storage and exchange of 3D city models and not for visualizing them. To visualize CityGML models over the web, they are usually converted to commonly used 3D graphics formats. We expect the CityGML iTINs ADE datasets to load faster over the web owing to their small file sizes and index‐based geometry representations. The CityGML iTINs ADE datasets can also be used for other applications utilizing CityGML models, such as noise modeling, flood modeling, visibility analysis, visualization for navigation purposes, and so on.
